# Contemporary Diagnostics of Cardiac Sarcoidosis: The Importance of Multimodality Imaging

**DOI:** 10.3390/diagnostics14171865

**Published:** 2024-08-26

**Authors:** Mihailo Stjepanovic, Filip Markovic, Ivan Milivojevic, Spasoje Popevic, Sanja Dimic-Janjic, Viseslav Popadic, Dimitrije Zdravkovic, Maja Popovic, Andrea Klasnja, Aleksandra Radojevic, Dusan Radovanovic, Marija Zdravkovic

**Affiliations:** 1 Clinic of Pulmonology, University Clinical Center of Serbia, 11000 Belgrade, Serbia; mihailostjepanovic@gmail.com (M.S.); flp.mark@gmail.com (F.M.); ivan.milivojevic94@gmail.com (I.M.); spasapop@gmail.com (S.P.); sanjadimicjanjic@gmail.com (S.D.-J.); 2 Faculty of Medicine, University of Belgrade, 11000 Belgrade, Serbia; zdravkovic.dika@gmail.com; 3 Department of Cardiology, University Clinical Hospital Center Bezanijska Kosa, 11000 Belgrade, Serbia; andrea.m93@gmail.com (A.K.); radaleksandra@gmail.com (A.R.); dusan_r@hotmail.com (D.R.); 4 Department of Radiology, University Clinical Hospital Center Bezanijska Kosa, 11000 Belgrade, Serbia; majapop89@gmail.com

**Keywords:** cardiac sarcoidosis, cardiovascular imaging, electrocardiography, echocardiography, cardiac magnetic resonance, positron emission tomography

## Abstract

Sarcoidosis is an inflammatory condition that can affect multiple organ systems and is characterized by the formation of non-caseating granulomas in various organs, including the heart. Due to suboptimal diagnostic rates, the true prevalence and incidence of cardiac sarcoidosis (CS) remain to be determined. In patients with suspected CS, an initial examination should include 12-lead ECG or ambulatory ECG monitoring, and echocardiography with the estimation of LV, RV function, and strain rate. In patients with confirmed extracardiac sarcoidosis and with high clinical suspicion for CS, sophisticated imaging modalities, including cardiac MRI and PET, are indicated. Typical inflammation patterns and myocardial scarring should pose a high suspicion for CS. In patients without diagnosed extracardiac sarcoidosis and high clinical suspicion, although with low diagnostic probability, an endomyocardial biopsy should be considered to establish the diagnosis of definite isolated cardiac sarcoidosis. Timely diagnosis enables the initiation of therapy and close monitoring of adverse cardiac events that can be life-threatening, including sudden cardiac death, ventricular tachycardia, high-degree AV block, and heart failure. Implementing biomarkers in correlation to cardiac imaging can determine the disease’s severity and progression but can also be helpful in following the treatment response. The formation of larger global registries can be helpful in the identification of independent predictors of adverse clinical events and the development of specific diagnostic algorithms to reduce the overall risk of this serious condition.

## 1. Introduction

The etiology and course of sarcoidosis remain largely unknown after more than a century since it was first described [1]. Sarcoidosis is an inflammatory condition that can affect multiple organ systems. It is characterized by the formation of non-caseating granulomas in various organs, resulting in decreased organ function [2]. The lungs, peripheral lymph nodes, central nervous system, skin, and the eye are most commonly involved [3].

Albeit rare, cardiac sarcoidosis (CS) is potentially life-threatening. Moreover, CS patients have a substantially impaired quality of life and worse clinical outcomes than patients with other sarcoidosis manifestations [4]. In CS, the formation of non-caseating granulomas can occur in all three layers of the heart although myocardial involvement is most prevalent.

The presentation of CS is heterogeneous. It may be asymptomatic but may also present as life-threatening arrhythmia, cardiomyopathy, heart failure, and even sudden cardiac death. This diverse presentation poses a great diagnostic challenge requiring specific diagnostic and prognostic algorithms.

## 2. Epidemiology

Distinct variations exist in the occurrence and frequency of sarcoidosis across various geographical regions and ethnic groups. Sarcoidosis demonstrates the highest incidence rates in Scandinavian countries, ranging from 11 to 24 cases per 100,000 individuals per year [5,6]. It is also prevalent among African Americans, with reported rates between 18 and 71 cases per 100,000 individuals annually [7,8]. In contrast, Asian countries exhibit the lowest incidence, with approximately 1 case per 100,000 individuals per year [9,10]. The average age of onset is 40–55 years of age, with a younger peak age at diagnosis in men (30–50 years of age) than in women (50–60 years of age) [11]. In Serbia, the estimated incidence of sarcoidosis is 16.5 per 100,000 individuals. A retrospective study conducted in Serbia, which included biopsy-proven sarcoidosis patients in the period from 2000 to 2023, highlighted the unique geographic and epidemiological distribution of sarcoidosis across the nation. This could indicate hereditary or environmental factors in the pathogenesis of sarcoidosis [12].

An autopsy study by Iwai K et al. found that CS may be more prevalent in Japan where it seemed to be the leading cause of death among sarcoidosis patients [13]. Clinical diagnosis of heart involvement occurs in only 5% of sarcoidosis patients. However, autopsy studies have detected cardiac involvement in up to 25% of sarcoidosis patients. In individuals with systemic sarcoidosis, the prevalence of cardiac involvement has ranged from 3.7% to 54.9% [14]. When cardiac involvement manifests as the first or sole organ manifestation, it suggests a more severe disease course compared to CS occurring in conjunction with extracardiac disease [15]. These findings suggest that due to suboptimal diagnostic rates, the true prevalence and incidence of CS remain to be determined. This is especially important as timely and accurate diagnosis facilitates appropriate intervention and better treatment outcomes in this patient population.

## 3. Risk Factors

Sarcoidosis shares a strong correlation with genetic factors, as well as environmental factors, including infectious agents and non-infectious antigens, metals, and combustible materials [16]. As already mentioned, the risk of sarcoidosis is higher in those of African or Scandinavian descent [5,6]. Potential infectious exposure to mycobacteria and Propionibacterium acnes, a skin commensal bacterium, is shown to be associated with sarcoidosis [17]. It has been demonstrated that smokers are less susceptible to sarcoidosis, probably due to the suppression of T-lymphocyte function and the phagocytic activity of macrophages [18]. Regarding cardiac sarcoidosis, older patients and those with diabetes mellitus and ischemic heart disease, have a higher risk of presenting with heart failure as a manifestation of CS [19]. 

## 4. Clinical Presentation

The clinical presentation of CS is heterogeneous. It can range from palpitations, syncope, orthopnea, dyspnea, and peripheral edema to sudden cardiac death. This variation of clinical presentation depends on the granulomas’ location within the heart and its extensivity. CS manifestations are commonly categorized into arrhythmic, cardiomyopathic, and pericardial groups [20]. However, it should be noted that up to 37% of sarcoidosis patients with cardiac involvement exhibit no symptoms [21].

### 4.1. Heart Failure

The prevalence of sarcoidosis-related cardiomyopathy is increasing. It is demonstrated that the 10-year incidence of clinical heart failure among patients with sarcoidosis is 3% [22]. Cardiomyopathy in patients with cardiac sarcoidosis can be presented as either heart failure with reduced or preserved ejection fraction or isolated right ventricular heart failure. In the early stages, reduced ventricular compliance and diastolic dysfunction can result in heart failure with preserved ejection fraction, while in severe cases can be presented as restrictive cardiomyopathy [23]. The presence of heart failure with reduced ejection fraction and RV involvement is associated with poor prognosis [24]. It is important to note that, although rare, isolated right ventricular cardiac sarcoidosis can phenotipically mimic arrhythmogenic right ventricular cardiomyopathy (ARVC) [25].

### 4.2. Atrial Arrhythmias

Atrial arrhythmias are present in 20–30% of patients with cardiac sarcoidosis [26]. The main pathophysiological mechanisms responsible for atrial arrhythmias are cardiac inflammation, granulomatous atrial involvement, and fibrosis, as well as left atrial remodeling [27]. Close monitoring, especially in symptomatic patients, and optimal anticoagulation threshold and rhythm control strategies are important parts of the management.

### 4.3. Ventricular Tachycardia

In previous observational studies, it has been shown that adverse events are mainly due to fatal ventricular arrhythmia events. Patients with low ejection fraction, high BNP levels, ventricular tachycardia or ventricular fibrillation history, and those requiring ablation to treat VT are at the highest risk of poor clinical outcomes [28]. Optimal diagnostic algorithms, especially in symptomatic patients, and timely prevention of sudden cardiac death, are of immense importance.

### 4.4. Conduction Disturbances

Conduction disturbances in patients with cardiac sarcoidosis, mainly bundle branch blocks and second or third-degree AV blocks are common, considering the predominant involvement of the interventicular septum [29]. In observational studies, up to 30% of patients with CS can have third-degree AV block, requiring permanent pacemaker implantation [30]. In those with heart failure symptoms and reduced ejection fraction with the indication for permanent pacemaker implantation, cardiac resynchronization therapy can be an important therapeutical modality to reduce heart failure hospitalizations and mortality. CRT-D should be considered in those with heart failure and ventricular arrhythmias [31].

## 5. Diagnosis

Diagnosing cardiac sarcoidosis (CS) remains challenging because it lacks specific biomarkers, particularly during the early stages of the disease [32]. Given the heterogeneous clinical manifestations (from asymptomatic to sudden cardiac arrest), establishing an early diagnosis of CS is essential [33]. 

Numerous international societies have provided criteria for the diagnosis of CS; however, the optimal diagnostic approach is still up for debate. Three main sets of clinical guidelines have been proposed by the Japanese Ministry of Health and Welfare (JMHW) [34], by the World Association for Sarcoidosis and Other Granulomatous Disorders (WASOG) [35], and in the consensus statement from the Heart Rhythm Society (HRS) [36]. 

The JMHW issued the first diagnostic guidelines for CS in 1993 [37] which were later revised in 2006 [38]. New guidelines included the utilization of novel non-invasive diagnostic methods. However, the 2006 JMHW diagnostic guidelines have not completely implemented the use of modern imaging modalities, such as FDG PET (not included in the diagnostic criteria) and cardiac magnetic resonance imaging (CMR) which was included, but as minor criteria. These modalities eventually demonstrated higher diagnostic accuracy in the clinical diagnosis of CS than the JMHW criteria [39,40,41]. The Japanese Circulation Society released revised guidelines in 2017, highlighting the importance of CMR and FDG-PET scans [34].

Societies in North America have created their own sets of CS diagnostic standards. The first algorithm was proposed by the US National Institutes of Health in 1999 [42], and it later served as the basis for the 2014 criteria created by the WASOG [43]. The WASOG criteria were partially referenced and built upon by a recent consensus statement from the HRS [42], ultimately providing a more modern set of clinical criteria for the diagnosis of CS. The American guidelines stress using contemporary imaging methods to diagnose CS. In a prospective cohort from 2017, where all patients had biopsy-confirmed extracardiac sarcoidosis and underwent CMR, the HRS criteria were demonstrated to detect more disease than the 2006 clinical criteria by JMWH [43]. The HRS has proposed the criteria for screening of CS with advanced cardiac imaging: suspected cardiac involvement in patients with biopsy-proven extracardiac sarcoidosis and one of the following symptoms (unexplained syncope/presyncope/significant palpitations lasting over 1–2 weeks) and/or abnormal ECG, and/or inconclusive echocardiogram results [18]. Additional details of the HRS and JCS criteria are shown in Table 1.

Novel position papers from the American Heart Association (AHA) and European Society of Cardiology (ESC) proposed a more integrated approach to reduce morbidity and mortality in patients with CS [44,45]. A recently published scientific statement from the American Heart Association provides an integrated framework for the diagnosing and managing of cardiac sarcoidosis. By integrating clinical data, laboratory parameters, and cardiac imaging findings, the scientific statement refers to the diagnosis of CS in a manner of the likelihood of cardiac sarcoidosis (definite, highly probable, probable, possible, low probability, unlikely) rather than in a binary fashion. The statement emphasizes the role of CMR and PET as mandatory in all patients with clinical suspicion of CS. This approach can stratify patients into several groups and provide timely management for certain groups and closer follow-up for others.

### 5.1. Electrocardiography

Even though considered neither sensitive nor specific enough to be a screening method for cardiac sarcoidosis, ECG is an integral part of sarcoidosis patient evaluation. In sarcoidosis patients, some ECG findings may hint towards potential cardiac involvement. While a normal ECG does not rule out cardiac sarcoidosis, it suggests that severe abnormalities are less likely present [46,47]. Among sarcoidosis patients, plenty of ECG findings have been reported. Most described conduction abnormalities and arrhythmias are right bundle branch block (RBBB), atrioventricular (AV) block of any degree, and ventricular tachycardia (VT) [48]. AV and BBBs arise due to sarcoid granuloma infiltration or consequent scarring of the interventricular septum, or involvement of the nodal artery that leads to ischemia in the conduction system. In cardiac sarcoidosis, VT occurs as a re-entry mechanism following granuloma scarring. These ECG findings also have a prognostic impact. Nordenswan et al. found that VT or AV block on ECG of sarcoidosis patients was associated with an increased risk of sudden cardiac death during a 5-year follow-up [49]. Sudden cardiac death due to AVB or VT accounted for 30–65% of sarcoidosis patients as per Roberts et al. [50]. The prognosis of sarcoidosis patients with cardiac involvement is more favorable in those that present with a high-degree AV block as opposed to VT and heart failure [51]. High-degree AV block is reversible in about 50% of cases when treated with steroids [52,53]. In untreated patients, however, it is not reversible. This may be due to the underlying pathophysiology of the condition mentioned above. When sarcoid granulomas, in the active phase of the disease, cause conduction abnormalities and not the consequent scarring, a response to steroid therapy could be expected. Therefore, early recognition of cardiac sarcoidosis and its treatment is paramount. The findings of AV block and bundle branch blocks in sarcoidosis patients should raise suspicion for cardiac involvement. On the other hand, in young and middle-aged patients with these ECG findings and complaints of presyncope and syncope, sarcoidosis should be considered as a differential diagnosis. Supraventricular arrhythmias, most commonly atrial fibrillation, have also been described in cardiac sarcoidosis [47]. They carry a more favorable prognosis than VT and are amenable to catheter ablation [54]. T-wave abnormalities were also observed in this patient population. According to Tanaka et al., T waves in avR in conjunction with bundle branch block were independently associated with cardiac involvement in sarcoidosis patients. Combined, these ECG abnormalities showed considerable diagnostic yield for cardiac involvement with a sensitivity of 94% and specificity of 89% [55]. However, these results have not been translated into everyday clinical practice due to study limitations, most notably the small sample size. The role of artificial intelligence and machine learning could be beneficial in identifying patients with a high probability of CS [56].

### 5.2. Echocardiography

**Figure 1:**
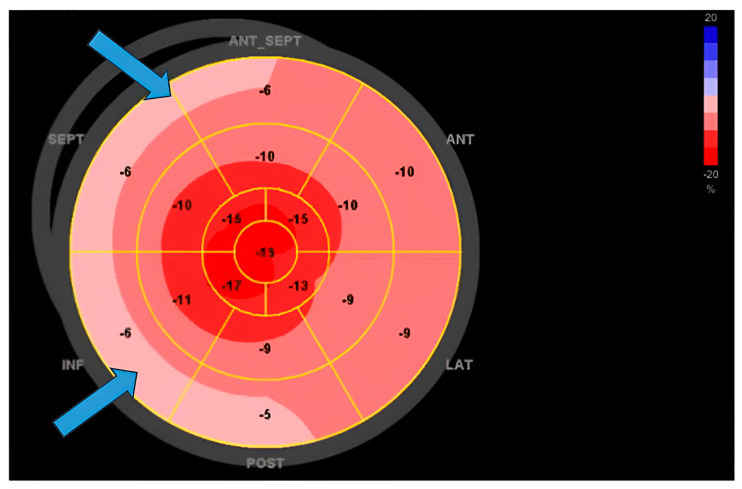
Myocardial strain in a patient with cardiac sarcoidosis and reduced ejection fraction: Reduced values of GLS predominantly in basal segments of the septum (marked with arrows).

Transthoracic echocardiography (TTE) stands out as the most accessible noninvasive method for cardiac imaging, offering crucial insights into cardiac structure and function. Echocardiographic abnormalities in sarcoidosis patients could raise suspicion of cardiac involvement. These include regional wall motion abnormalities, regional wall thickening, and valvular dysfunction, myocardial echogenicity, and RV free-wall aneurysm formation [57]. Such findings, however, are not sufficient to establish a diagnosis of CS but should trigger further diagnostic procedures utilizing more sensitive modalities such as positron emission tomography (PET) or cardiac magnetic resonance (CMR) as per the Heart Rhythm Society (HRS) consensus. The same consensus document suggests that TTE should be used as a first-line screening tool for diagnosing CS given its widespread availability [18]. Certain echocardiographic findings may have prognostic value. The existence of basal septal thinning at the time of CS diagnosis is correlated with adverse outcomes such as increased mortality, occurrence of ventricular arrhythmias, and hospitalization due to heart failure. This association remains significant irrespective of corticosteroid therapy or cardiac resynchronization therapy [58]. A more recent study found that basal septal thinning is associated with the future development of left ventricular systolic dysfunction even when the function of the left ventricle is preserved at the time of CS diagnosis [59]. Left ventricular ejection fraction (LVEF) remains a critical echocardiographic parameter in the evaluation of a patient with CS, as it can provide valuable insights into the long-term outcome of the condition. Individuals diagnosed with cardiac sarcoidosis and presenting with severe left ventricular systolic dysfunction (LVEF ≤ 35%) or moderate dysfunction (LVEF 36–50%) face worse prognosis compared to those with preserved LVEF (>50%). Although patients with LVEF ≤ 35% may witness some left ventricular recovery following immunosuppression, the majority tend to persist in the severely impaired category [60]. Novel echocardiographic parameters such as global longitudinal strain (GLS) have shown potential both in screening for CS and monitoring of these patients. Patients with systemic sarcoidosis have been found to exhibit significantly more impaired GLS [61,62]. Murtagh et al. found that GLS was significantly reduced in CS patients even with preserved LVEF using a cutoff of 17% [63]. The strain has been proposed as a more sensitive measure of subclinical myocardial dysfunction than ejection fraction [57]. It is important to keep in mind that a wide overlap of standard deviations between diseased and healthy subjects has also been reported. Considering that reference GLS ranges and cut-off values vary with different vendors and software and that the majority of patients initially have subtle changes, GLS values can be difficult to interpret and should be considered with other parameters and diagnostic findings. On the other hand, low GLS in CS patients has been associated with poor prognosis and outcomes including heart failure-related hospitalizations, need for device therapy, arrhythmias, and all-cause mortality (Figure 1) [64,65]. These findings support the notion that GLS measurements should be incorporated into the TTE protocol, and patients with low GLS should be closely monitored.

### 5.3. Endomyocardial Biopsy (EMB)

**Figure 2:**
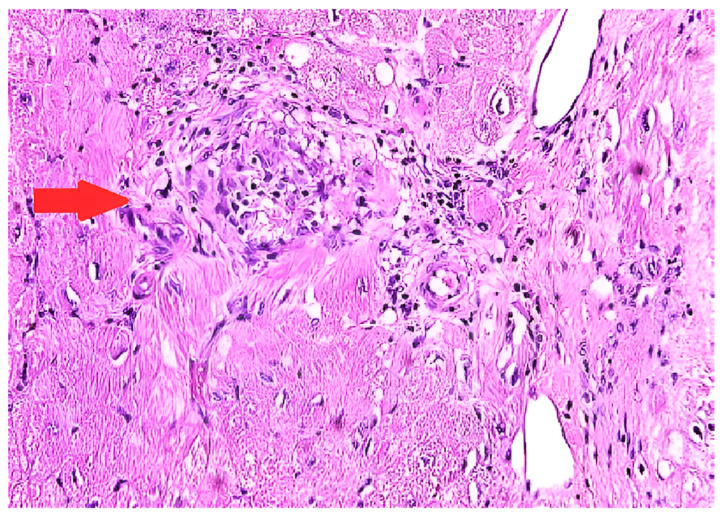
Endomyocardial biopsy in a patient with cardiac sarcoidosis reveals a non-necrotizing granulomatous inflammation (marked with arrow) with patchy interstitial fibrosis on hematoxylin and eosin staining.

The most definitive way of diagnosing CS is through EMB, which shows histologic noncaseating granuloma with other causes excluded (Figure 2) [66]. The sensitivity of EMB for diagnosing CS was evaluated in a study that included 851 patients, and it demonstrated the sensitivity of EMB around 20% [67]. Several other studies showed similar results [68,69]. The low sensitivity of EMB can be partially explained by the patchy involvement of the myocardium [70]. Higher sensitivity can be achieved with intracardiac voltage mapping or imaging-guided EMB [71]. A study from Japan revealed higher positivity of EMB in patients with reduced left ventricular ejection fraction [72]. This could suggest that patients with severe cardiac injury and widespread disease activity are more likely to receive a histological diagnosis via EMB due to multifocal or diffuse changes.

### 5.4. Cardiac Magnetic Resonance (CMR)

**Figure 3:**
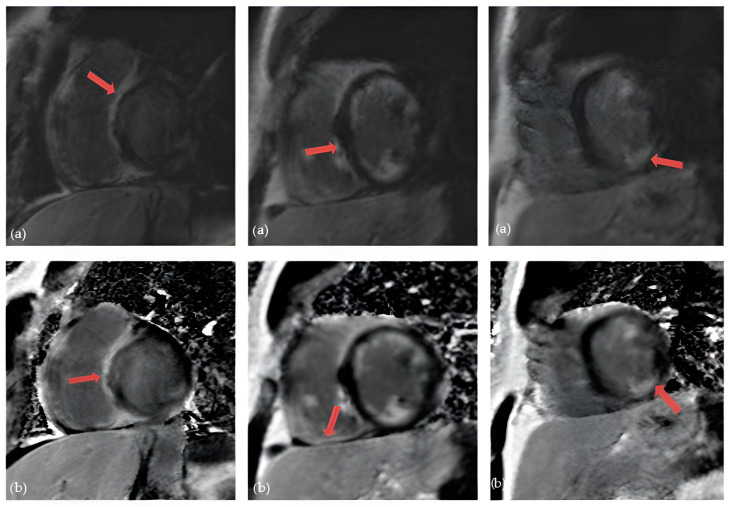
Cardiac magnetic resonance in a patient with cardiac sarcoidosis: (**a**) LGE MAG study, short axis: LGE in septum and inferior segments (marked with arrows); (**b**) LGE PSIR study, short axis: LGE changes in left and right ventricle, predominantly in septal segments (marked with arrows) (Avanto MRI, Siemens Healthcare GmbH, Erlangen, Germany, 1.5 T, CMR Lab University Clinical Hospital Center Bezanijska kosa, Belgrade, Serbia).

Over the last few years, advanced cardiac imaging which includes CMR has become the preferred way of diagnosing CS due to its non-invasiveness. [19]. It can identify a wide range of myocardial abnormalities in patients with CS, including inflammation, fibrosis, reactive edema, and granulomatous infiltration. Kouranos et al. recently demonstrated that CMR has a significantly greater sensitivity in identifying cardiac involvement in relation to echocardiography: 97% vs. 27%, respectively [43]. Conversely, in a study that analyzed explanted hearts, CS was histologically confirmed in only 1 of 8 cases presumed to have CS by CMR [73]. The moderately low specificity of CMR presented the biggest challenge to the diagnosis of CS [74]. However, the specificity of CMR in diagnosing CS increased with the development of novel tissue characterization sequences. This was shown in a large meta-analysis from 2018 that included 649 patients. One group included studies between 2005 and 2011 where the overall sensitivity was 91% and specificity 80%. The second subgroup included studies between 2011 and 2017 where the overall sensitivity was 95% and specificity was even 92% [75]. The hallmark of CS on CMR is the presence of late gadolinium enhancement (LGE) [76]. The main principle of CMR is based on the washout of gadolinium, which is slower in edematous, inflammatory, and scarred tissues, making it visible in CMR’s delayed images [19]. The presence of LGE is not pathognomonic for CS, as it can be seen in a variety of nonischemic cardiomyopathies. However, certain patterns of cardiac involvement are thought to be typical of CS [77]. A systematic review from 2019, which included patients with histologically proven CS, observed that the most prevalent pattern of LGE is mid-wall or sub-epicardial enhancement in the septum, lateral wall, and basal ventricular wall (Figure 3). However, there have also been reports of subendocardial or transmural augmentation in other myocardial regions [78].

Additionally, a large meta-analysis demonstrated that the presence of LGE on CMR has important predictive values in patients with CS, with an increased tendency toward adverse events, including overall mortality and heart failure hospitalization [79]. 

**Figure 4:**
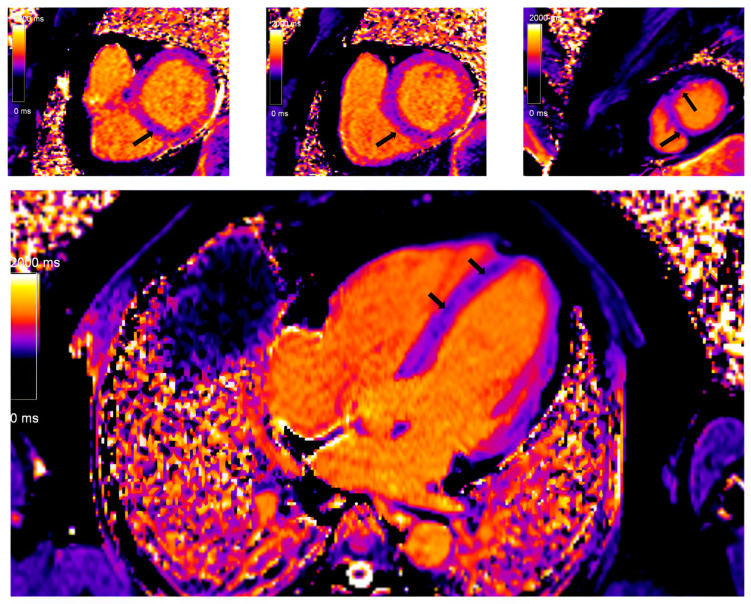
Myocardial tissue mapping in a patient with cardiac sarcoidosis and high degree AV block: native T1 mapping revealing the areas of shortened native T1 time indicating myocardial fibrosis predominantly in the septum (marked with arrows) (Avanto MRI, Siemens Healthcare GmbH, Erlangen, Germany, 1.5 T, CMR Lab University Clinical Hospital Center Bezanijska kosa, Belgrade, Serbia).

In recent years, with hardware and post-processing software improvements predominantly driven by multiparametric tissue mapping, the diagnosis and risk stratification of patients with CS has improved. Regional T2 mapping values are independent predictors of active myocardial inflammation in CS and may add additional discriminatory capability [80]. Prolonged or shortened native T1 time, both focally in the septum and globally, is a significant marker of the severity of the disease. At the same time, the fraction of extracellular volume (ECV) is an independent predictor of future serious adverse events among patients with CS (Figure 4) [81]. The ECV estimation is also important in differentiating other cardiac conditions, predominantly myocarditis. It is important to note that CS patients had higher ECV values in the areas with the LGE phenomenon compared to patients with myocarditis.

### 5.5. ^18^Fluorodeoxyglucose Positron Emission Tomography (FDG-PET)

**Figure 5:**
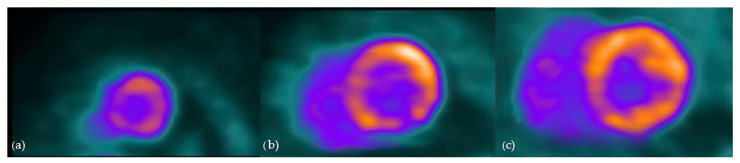
Cardiac PET in a patient with cardiac sarcoidosis: ^18^F-FDG uptake in most of left ventricle, consistent with active inflammation; No significant uptake in apex or mid-inferolateral segment, compatible with possible fibrosis ((**a**)—apex, (**b**)—mid-ventricle, (**c**)—basal).

Cardiac FDG-PET imaging is used in cases of suspected CS since it can identify glucose uptake by inflammatory cells in sarcoid granulomas [82]. According to the Japanese Society of Nuclear Cardiology guidelines from 2019, it is recommended that a low-carbohydrate, high-fat diet followed by fasting for a minimum of 12 h be used to inhibit the physiologic glucose metabolism of the heart to enable diagnostic imaging [83]. Importantly, 25% of cardiac PET scans are not diagnostic because the physiologic glucose uptake is not sufficiently suppressed [84]. A meta-analysis from Ontario using the JMHW guidelines as the reference standard for diagnosing CS reported a sensitivity of 89% and specificity of 78% for FDG PET [85]. Those values are genuinely equivalent to the CMR’s sensitivity (75–100%) and specificity (76.9–78%), according to some authors [86]. The JMHW criteria used in this meta-analysis have certain drawbacks, such as the need for extracardiac sarcoidosis in the diagnostic criteria and insufficient validation. Furthermore, these clinical criteria are insufficient to diagnose isolated CS, a well-described clinical entity that may arise in about 25% of cases [87,88]. When a patient is adequately prepared, the normal FDG-PET shows no myocardial FDG uptake, while the predominant pathological FDG uptake patterns are focal or focal on diffuse imaging patterns [89]. Although any part of the left ventricle can be involved, the most common place for CS to occur is the basal segments [42]. Besides the left ventricle, assessing regions of localized FDG uptake in the right ventricle is particularly important, since this could be linked to a less favorable outcome (Figure 5) [89].

Additionally, with FDG-PET, it is also possible to quantify the degree of inflammation and evaluate the outcome of anti-inflammatory treatment. According to Osborne and colleagues, a significant improvement in left ventricular ejection fraction was linked to a decrease in the intensity and extent of myocardial inflammation as determined by FDG-PET in 23 patients who had serial scans [90]. In a retrospective study by Blankstein and colleagues, where 118 patients with confirmed or suspected CS were assessed, it was demonstrated that focal FDG uptake on cardiac PET indicated individuals at higher risk of mortality or ventricular tachycardia [41]. The results of a recent meta-analysis indicate a possible role for FDG-PET in the prognosis of CS. In this study, which included 515 patients, the presence of abnormal FDG-PET pattern, especially considerable right ventricle uptake, indicated a higher risk of severe cardiac events [91]. Gowani et al. published some conflicting data where FDG uptake did not contribute to prognostic value [92].

**Figure 6:**
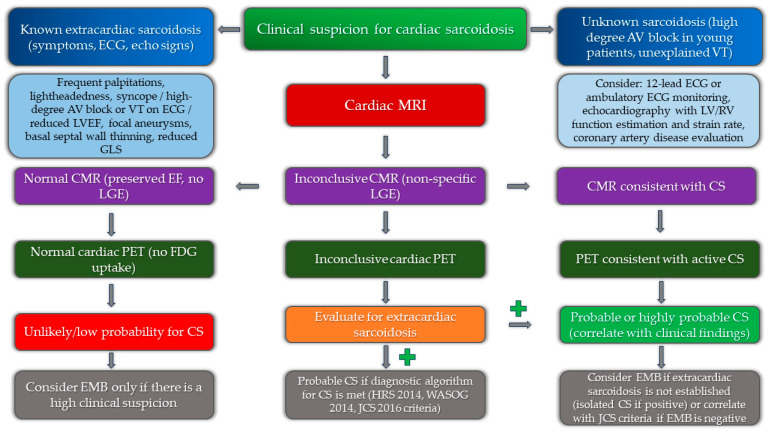
Implementation of multimodality imaging into diagnostic algorithm for the diagnosis of cardiac sarcoidosis.

The role of multimodality imaging as a part of the diagnostic algorithm is presented in Figure 6.

### 5.6. The Role of Cardio-Specific Biomarkers in Patients with Cardiac Sarcoidosis: Correlation with Imaging Findings

Biomarkers can play an important diagnostic and prognostic role in patients with cardiac sarcoidosis. Among routinely used biomarkers, it was shown that troponin T, NT-proBNP, and creatinine could predict clinically significant outcomes in patients with CS [93]. These markers could indicate the disease severity and progression but also be helpful in following the treatment response. ACE levels correlate well with LGE registered on cardiac magnetic resonance, while 1,25-OHVit-D levels correlate with FDG-PET activity [94]. Modified diagnostic algorithms were developed to screen for cardiac involvement in patients with sarcoidosis effectively. Kumar et al. incorporated contemporary echocardiographic parameters and cardiac biomarkers (NT-proBNP and troponin levels) into the Heart Rhythm Society (HRS) diagnostic algorithm and increased the yield of detecting cardiac involvement by 30% [95]. Novel markers include circulating miR-126 and miR-223 microRNAs that are significantly up-regulated in patients with cardiac sarcoidosis and correlate well with CMR and PET findings [96].

## 6. Conclusions

Cardiac sarcoidosis has a higher prevalence than previously thought, mainly due to the improvement of modern imaging techniques and their capability to detect subtle changes. Despite significant recent advances, the diagnosis of CS still poses a challenge. Simplifying diagnostic criteria and developing new procedures is crucial not only in the diagnostic process but also in stratifying patients and following therapeutic modalities’ potential benefits. At the same time, additional work is needed in the field of diagnosing cases of isolated CS. Nonetheless, new imaging techniques and modalities have significantly increased global awareness of CS. Considering the variety of clinical presentations and multi-modality imaging findings, implementing of artificial intelligence can provide diagnostic and prognostic advantages and, possibly, therapeutic monitoring. The formation of larger global registries can also be helpful in the identification of independent predictors of adverse clinical events and the development of specific diagnostic algorithms to reduce the overall risk of this severe condition.

## Figures and Tables

**Table 1:** Japanese cardiac sarcoidosis and Heart Rhythm Society guidelines (Terasaki et al., 2017 [35]; Birnie et al., 2017 [32]).

2017 JCS Guideline on Diagnosis and Treatment of Cardiac Sarcoidosis	2014 HRS Expert Consensus Statement on the Diagnosis and Management of Arrhythmias Associated with Cardiac Sarcoidosis
Histologic diagnosis Clinical diagnosis group when extracardiac sarcoidosis is confirmed and two or more major criteria or one major and two or more minor criteria are fulfilled Major criteria High-grade atrioventricular block (including complete atrioventricular block) or fatal ventricular arrhythmia (e.g., sustained ventricular tachycardia, and ventricular fibrillation) Basal thinning of the interventricular septum or abnormal ventricular wall anatomy (ventricular aneurysm, thinning of the middle or upper ventricular septum, regional ventricular wall thickening) Left ventricular contractile dysfunction (left ventricular ejection fraction less than 50%) or focal ventricular wall asynergy 67 Ga citrate scintigraphy or FDG PET reveals abnormally high tracer accumulation in the heart Gadolinium-enhanced MRI reveals delayed contrast enhancement of the myocardium Minor criteria Abnormal ECG findings: Ventricular arrhythmias (nonsustained ventricular tachycardia, multifocal or frequent premature ventricular contractions), bundle branch block, axis deviation, or abnormal Q waves Perfusion defects on myocardial perfusion scintigraphy (SPECT) Endomyocardial biopsy: Monocyte infiltration and moderate or severe myocardial interstitial fibrosis	Histologic diagnosis Clinical diagnosis of probable CS is present if there is extracardiac sarcoidosis confirmed and one or more of the following: Unexplained reduced LVEF (<40%) Corticosteroid- and/or immunosuppressant-responsive cardiomyopathy or heart block Unexplained sustained (spontaneous or induced) ventricular tachycardia Mobitz type II second-degree AV block or third-degree AV block Patchy uptake of cardiac FDG-PET in a pattern consistent with cardiac sarcoidosis Late gadolinium enhancement on CMRI in a pattern consistent with cardiac sarcoidosis Positive gallium uptake in a pattern consistent with cardiac

## Data Availability

Not applicable.
